# Post-Traumatic Cilia Remaining Inert in the Posterior Chamber for 50 Years

**DOI:** 10.3390/diagnostics13091575

**Published:** 2023-04-28

**Authors:** Cosmin Adrian Teodoru, Mihai Dan Roman, Adrian Hașegan, Claudiu Matei, Cosmin Mohor, Mihnea Munteanu, Mihaela Laura Vică, Horea Vladi Matei, Horia Stanca, Maria-Emilia Cerghedean-Florea, Horațiu Dura

**Affiliations:** 1 Faculty of Medicine, “Lucian Blaga” University of Sibiu, 550024 Sibiu, Romania; 2 Department of Ophthalmology, “Victor Babes” University of Medicine and Pharmacy, 300041 Timisoara, Romania; 3 Department of Cellular and Molecular Biology, “Iuliu Haţieganu” University of Medicine and Pharmacy, 400012 Cluj-Napoca, Romania; 4 Institute of Legal Medicine, 400006 Cluj-Napoca, Romania; 5 Department of Ophthalmology, “Carol Davila” University of Medicine and Pharmacy, 050474 Bucharest, Romania

**Keywords:** intraocular foreign body injuries, intraocular cilia, COVID-19

## Abstract

Intraocular foreign body injuries (IOFB) can lead to a number of intraocular pathologies; the visual results depend on the mechanism of the injury, the type of foreign body and the subsequent complications. The presence of intraocular cilia (eye lashes) following penetrating injury or surgical intervention is uncommon. In the present paper, we present a case of a 58-year-old woman with a history of eye trauma and a perforated corneal wound in the left eye that occurred 50 years ago. On the ophthalmological exam we noticed in the anterior chamber a straight linear extension, resembling cilia, extending behind the iris. The patient reports that it appeared during COVID-19 infection, after repeated episodes of coughing. After a follow-up period, we decided to remove the eyelash; 24 h after surgery, the patient complained of severe eye pain. Intraocular pressure (IOP) in LE was 54 mmHg. The slit-lamp examination showed perikeratic congestion, corneal edema and mydriasis. Eye hypotensive treatment was started immediately and the patient’s general condition slightly improved. Intraocular cilia can be tolerated for many years without causing any ocular reaction. The decision for surgical intervention must be taken according to the individual needs of the patient and his ocular characteristics with careful pre- and post-operative follow up.

**Figure 1:**
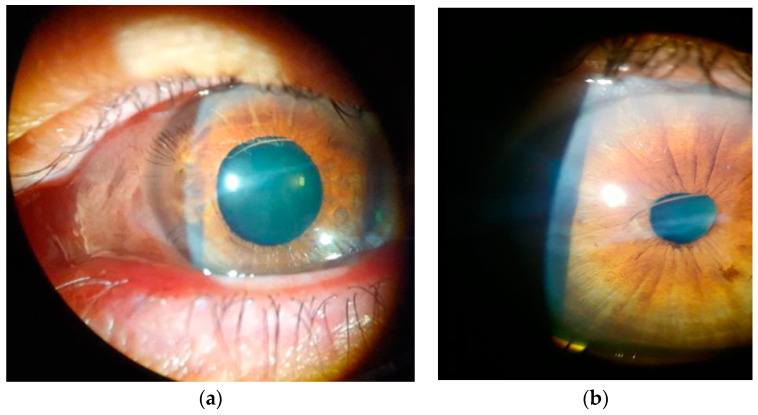
Slit-lamp examination of a 58-year-old woman with a history of eye trauma and a perforated corneal wound, sutured approximately 50 years ago in the left eye. She presented in our department accusing strange shadows in the left visual field. The ophthalmological exam revealed a visual acuity (BCVA) for the left eye—0.1 (decimal notation) and the intraocular pressure measured with the I-care ic200 tonometer (I-Care Finland Oy, Helsinki, Finland) of 14 mmHg. Examination of the anterior segment of the left eye revealed a central corneal leukoma and a pupil with diminished pupillary light reflex compared to the contralateral eye. In the anterior chamber (AC) a straight linear extension, resembling cilia, extending behind the iris in the upper part of the pupil was noted (**a**,**b**). The anterior chamber was quiet with no cells or flare and no posterior synechia. The posterior pole was normal. The patient reports that it appeared during infection with COVID-19, after repeated episodes of coughing. The BCVA of the other eye was 1 (decimal notation). The IOP was 14 mmHg, clear cornea, normal anterior chamber, regular, central, reflexive pupil. Slit-lamp examination of the LE (Figure 1) suggested that the wondering shadow was caused by an intraocular eyelash. Our hypothesis was that the eyelash entered her eye 50 years ago and remained silent until the coughing caused by COVID-19 infection mobilized it, bringing an end of it in the pupillary aria. She denied pain or any changes in visual acuity. There were no remarkable medical or ocular histories. The patient denied any history of eye surgery or trauma except from childhood. She was not taking any medications and had no known drug allergies. The presence of an intraocular eyelash is rare and unusual, with these cases representing approximately 0.4% of all cases of intraocular foreign bodies. Most of the time, the eyelash is the consequence of a perforating eye trauma or it can appear post-surgically [1]. The most frequent locations reported were the anterior segment of the eye, but, extremely rarely, eyelashes were also found in the vitreous or the retina [1,2]. The inflammatory reaction in these particular cases is unpredictable. While in some cases a series of serious eye complications can occur with the potential of vision loss, others remain totally asymptomatic for a long period of time [3,4,5,6]. Initially, we followed the patient for several months before surgery. Unfortunately, her condition remained unchanged and, according to the patient, her quality of life was affected by the presence of the “shadow”. During all this period, intraocular pressure was normal; no signs of ocular inflammation were noticed. Preoperatively, a series of imaging investigations were performed (Figure 2 and Figure 3).

**Figure 2:**
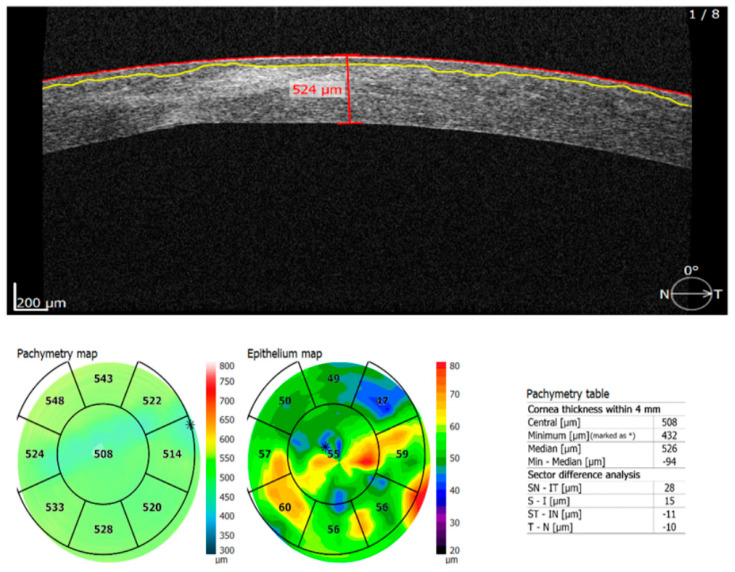
Central thickness of the cornea was measured using optical coherence tomography (Copernicus REVO SOCT (Optopol Technology (Zawiercie, Poland)). We noticed a smaller corneal thickness (508 µm) and a slightly irregular appearance of the central cornea most likely due to old surgery.

**Figure 3:**
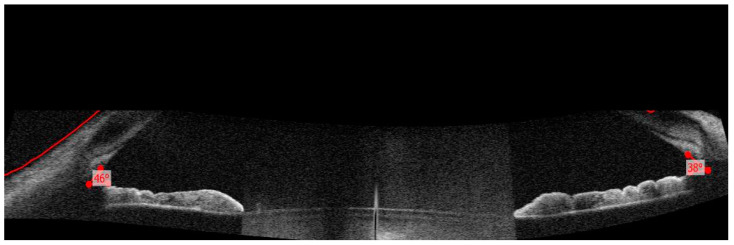
We also performed an irido-corneal angle measurement using optical coherence tomography (OCT-Copernicus REVO SOCT (Optopol Technology (Zawiercie, Poland)). The measurements were made manually on these images. We noticed an open iridocorneal angle in the temporal (46°) and nasal quadrants (38°), respectively. Additionally, the patient performed a series of routine tests such as complete blood count, biochemistry (urea, creatinine, glucose, amylase, AST, ALT), coagulogram (APTT, PT, INR), and inflammatory tests (C-reactive protein, VSH, fibrinogen). All were within normal limits with no evidence of general inflammation. The surgery was performed without any special incidents (Figure 4).

**Figure 4:**
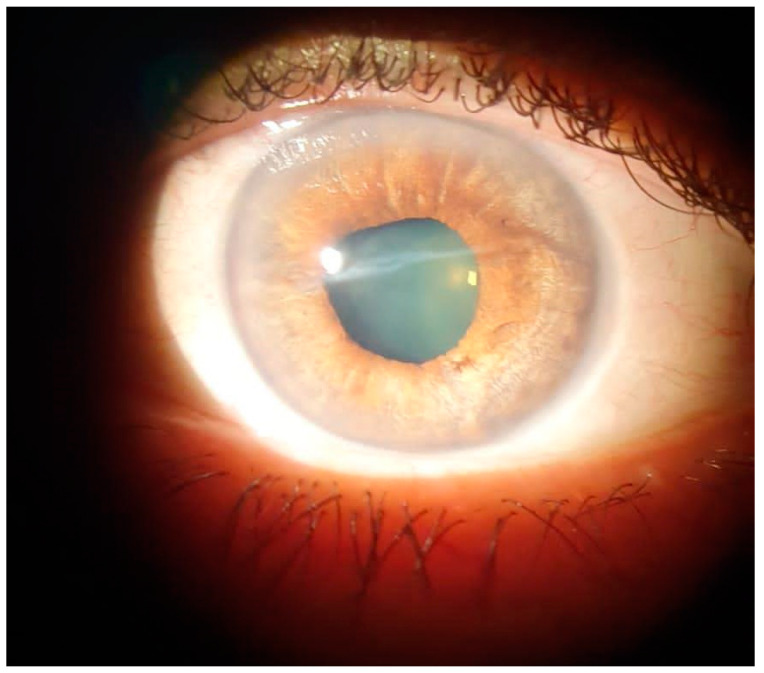
Slit-lamp examination on left eye—2 h postoperatively. Ophthalmic drops for mydriasis and topical anesthesia were first administered. A 2.2 mm corneal incision at 12 o’clock was performed. Some viscoelastic substance was injected in the anterior chamber to maintain the chamber. The extension was easily mobilized using capsulorhexis forceps with gentile movements. No hemorrhage occurred in the chamber. After the extraction, the viscoelastic substance was removed by manual irrigation-aspiration. A subconjunctival injection with Gentamycin and Dexamethasone was performed after the surgery. Postoperatively, the patient received topical treatment with chloramphenicol 0.25%/betamethasone 0.1% (Betabioptal; Laboratoires Théa, France) 4 times a day. The extension appeared to be a 6 mm-long cilium (Figure 5a), the nature of which was confirmed by histopathological examination (Figure 5b).

**Figure 5:**
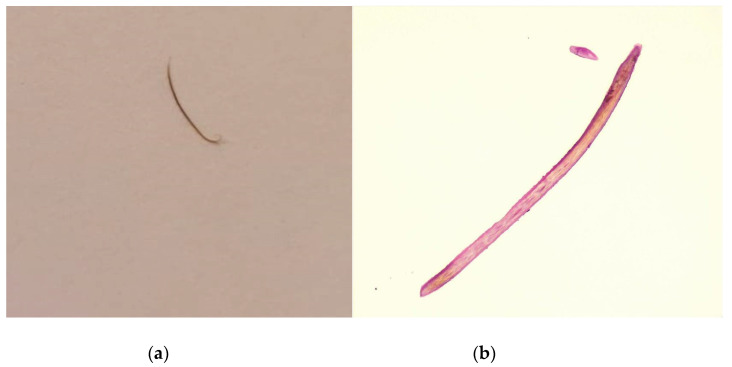
Postoperative aspect (**a**) and histopathological view (**b**) of the eyelash. Then, 24 h after surgery, the patient complains of severe eye pain, congestion, nausea and vomiting. The slit-lamp examination shows perikeratic congestion, corneal edema, slowly reflexive pupil, mydriasis and an intraocular pressure of 54 mmHg. Eye hypotensive treatment topical drops (Dorzolamide, Timolol) and systemic medication (Mannitol, Acetazolamide) was started immediately. Further, 72 h after the surgery the patient’s general condition did slightly improved, with a decrease in symptoms. Intraocular pressure on LE was 8 mmHg. The conjunctival congestion and the corneal edema were in remission. We have followed the patient periodically since the surgery. Intraocular pressure has been maintained at normal values, with no signs of inflammation in the AC. Visual acuity remains the same, but the patient has had no other subjective complaints (shadows). Perforating eye wounds associated with the presence of an eyelash in the anterior or posterior segment are rare and unusual. There are few cases described in the scientific literature. Generally, the cause is known or associated with an event, traumatic or a surgical intervention, but there are also situations with unknown etiology [7,8]. The response of the eye to intraocular eyelashes is variable. The eyelash may be symptomatic, intra-ocular cilia can be associated with corneal edema, corneal graft rejection, granulomatous or non-granulomatous iridocyclitis, cyst formation, lens abscess, retinal detachment and endophthalmitis or may remain asymptomatic for long periods [3,4,7,9,10]. The asymptomatic course of intra-ocular cilia is related to its relatively inert nature compared to other organic materials and the immune privileged feature of the eye [3]. In the presented case, the presence of the intraocular eyelash is closely related to the childhood trauma and remained asymptomatic for over 50 years. In such cases, the management is controversial. While some authors recommend careful follow up, others recommend the removal of the intraocular foreign body and the reduction of the inflammatory potential [11]. There are certain factors that must be taken into account when deciding to extract a latent foreign body, such as the location and size of the initial wound, the position of the cilia in relation to the anterior or posterior segment, the possibility of creating additional intraocular lesions or affecting the postoperative visual acuity [12]. Although the extraction of the foreign body proceeded without incidents, postoperatively there was a strong ocular response, represented by a sudden increase in intraocular pressure. The cause remains controversial, if the increased pressure was associated strictly to the viscoelastic substances used or also due to an exaggerated local inflammatory response caused by the eyelash extraction [13]. To conclude, intraocular cilia can be tolerated for many years without causing any ocular reaction. However, the management of such cases remains controversial. In our case, the decision of the surgical intervention, after several years from the incident, was taken according to the individual needs of the patient (improving the patient’s symptomatology and quality of life) and his ocular characteristics. Periodic monitoring is, in any case, mandatory.

## Data Availability

All relevant data have been presented in this manuscript, and further inquiry can be directed to the corresponding author.
